# PI3K signaling in breast cancer: therapeutic advances versus adaptive resistance

**DOI:** 10.1172/JCI207635

**Published:** 2026-10-15

**Authors:** Fabiana Napolitano, Nisha Unni, Carlos L. Arteaga

**Affiliations:** 1 Department of Clinical Medicine and Surgery, University of Naples Federico II, Naples, Italy.; 2 UT Southwestern Simmons Comprehensive Cancer Center and; 3 Department of Internal Medicine, UT Southwestern Medical Center, Dallas, Texas, USA.

## Abstract

Somatic alterations in the phosphoinositide 3-kinase (PI3K) pathway are frequent oncogenic drivers in cancer and are particularly relevant in estrogen receptor–positive breast cancer, where PI3K signaling integrates hormonal, growth factor, and metabolic inputs. Therapeutic targeting of PI3K has been limited by toxicity, pathway crosstalk, and adaptive resistance. Recent advances in structural and functional characterization of *PIK3CA* mutations have enabled the development of more selective inhibitors, including PI3K isoform–selective and emerging allosteric PI3Ka mutant–targeted compounds. These approaches aim to improve the therapeutic index while maintaining pathway inhibition. In parallel, combination strategies with endocrine therapy and CDK4/6 inhibitors have demonstrated clinical benefit in selected patient populations. However, responses are often transient owing to compensatory signaling and pathway reactivation. Additional metabolic and transcriptional adaptations further contribute to sustaining tumor cell survival. Overall, PI3K-targeted therapy is evolving along two complementary paths: increased mutant-selective inhibition to improve therapeutic index and broader pathway-level strategies designed to overcome adaptive resistance and achieve more durable responses. Ongoing efforts focus on optimizing combination strategies, enhancing development of predictive biomarkers, and improving therapeutic sequencing to achieve durable responses while minimizing toxicity.

## Introduction

Somatic alterations in the phosphoinositide 3-kinase (PI3K) signaling pathway are oncogenic drivers in solid tumors and hematologic malignancies as well as in nonmalignant overgrowth syndromes (1–4). Analysis of the AACR Project GENIE cohort v19.0 (5) shows that alterations in *PIK3CA*, encoding the p110α catalytic subunit of PI3K, are among the most common genomic alterations across solid tumors, occurring, e.g., in nearly 50% of endometrial cancers and approximately 35% of breast cancers (5–7).

Despite their high prevalence, *PIK3CA* mutations are considered weaker oncogenic drivers compared with canonical alterations such as those in *KRAS* or *EGFR* (6). While they promote cell growth, survival, and proliferation, *PIK3CA* mutations are usually insufficient on their own to induce full malignant transformation and typically cooperate with additional genetic and epigenetic events during tumor evolution. This limited transforming potential is reflected by mosaic human developmental disorders caused by postzygotic *PIK3CA* mutations, collectively known as *PIK3CA*-related overgrowth spectrum (PROS) (8). PROS include a wide range of syndromes characterized by segmental tissue overgrowth and vascular malformations (7) but with no substantial increase in cancer risk (8, 9), likely due to the absence of the cooperative secondary mutations required for cancer transformation, such as *TP53* inactivation (6, 7). Conversely, germline mutations in other tumor suppressor genes can increase risk of developing cancers, such as loss of function of the lipid phosphatase and tensin homolog *PTEN*, which causes PTEN hamartoma tumor syndrome, characterized by multiple hamartomas and markedly increased lifetime risks of breast, endometrial, thyroid, and other malignancies (10).

Among solid tumors, breast cancer is a paradigmatic example of *PIK3CA*-driven malignancy, where *PIK3CA* mutations arise early during tumor evolution and constitute one of the most clinically relevant therapeutic targets. *PIK3CA* mutations are reported in 30%–40% of primary breast tumors and retained at similar or even higher frequencies in relapsed and metastatic disease (11). Although present in all molecular subtypes, their prevalence and biological implications differ according to tumor subtype: *PIK3CA* mutations are reported in approximately 10%–15% of triple-negative breast cancers, with particular enrichment in the luminal androgen receptor subtype (12); approximately 20%–30% of human epidermal growth factor receptor 2–positive (HER2^^+^^) breast tumors, in which *PIK3CA* mutations are associated with reduced sensitivity to anti-HER2 therapies (13, 14); and approximately 35%–45% of luminal A/B hormone receptor–positive/HER2^^–^^ (HR^^+^^/HER2^^–^^) breast cancers (15). In HR^^+^^ breast cancers, *PIK3CA* mutations are particularly relevant owing to the bidirectional crosstalk with estrogen receptor (ER) signaling (16, 17), promoting tumor growth and endocrine resistance. This biological interaction has informed combinations of PI3K inhibitors with anti-ER therapies and may explain why PI3K inhibitors were first approved for patients with HR^^+^^ breast cancer rather than for other malignancies harboring PI3K pathway alterations. Furthermore, the use of biomarker-selected patient populations and rational combination strategies with endocrine therapy has enabled PI3K inhibition to achieve meaningful clinical benefit in this disease.

Despite the compelling biological rationale and the high frequency of PI3K pathway alterations, translating PI3K inhibition into durable clinical benefit has proven challenging. Therapeutic targeting of PI3K has been limited by toxicity, adaptive resistance mechanisms, and context-dependent pathway rewiring. Addressing these challenges has required a deeper understanding of PI3K biology at both the molecular and structural levels. Recent advances in structural biology have revealed how *PIK3CA*-activating mutations alter kinase conformation and signaling dynamics, thus reshaping therapeutic strategies and enabling the development of a novel class of inhibitors (18–20).

Herein, we focus on the recent advances in our understanding of the PI3K pathway, discussing both the opportunities for therapeutic intervention and the challenges that have emerged for clinical translation.

## Overview of PI3K signaling

PI3Ks are lipid kinases that translate extracellular stimuli into intracellular programs regulating proliferation, survival, metabolism, and cell fate (21).

### PI3K isoforms and activation.

Based on structural organization and substrate specificity, PI3Ks are classified into three classes. Class I PI3Ks are further subdivided into class IA and IB, each consisting of a p110 catalytic subunit associated with a regulatory subunit. In the human genome, class IA PI3Ks comprise three catalytic isoforms — p110α (encoded by the *PIK3CA* gene), p110β (*PIK3CB*), and p110δ (*PIK3CD*) — and five regulatory isoforms or splice variants: p85α, p85β, p55γ, p55α, and p50α. Upon activation, class I PI3Ks catalyze the generation of phosphatidylinositol 3,4,5-trisphosphate (PIP3) from PIP2. PIP3 is the main second messenger generated by PI3K. At the plasma membrane, PIP3 acts as the docking site for several pleckstrin homology domain–containing signaling proteins, such as phosphoinositide-dependent kinase-1 (PDK1) and AKT (PKB) (22, 23). The lipid phosphatase PTEN antagonizes the function of PI3K by dephosphorylating PIP3 back to PIP2 (7, 8). Upon PIP3-mediated recruitment to the plasma membrane, AKT is phosphorylated at Thr308 by PDK1 and at Ser473 by mTOR complex 2 (mTORC2), achieving full activation (24). Activated AKT phosphorylates multiple substrates that regulate protein synthesis, glucose metabolism, apoptosis, and cell survival, including TSC2, PRAS40, FOXO, and others (25) (Figure 1A).

### ERα/MAPK signaling pathways crosstalk.

In ER^^+^^ breast cancer, PI3K signaling operates as a central signaling hub that integrates ERa activity with growth factor, metabolic, and stress-response pathways. This integration relies on bidirectional crosstalk between PI3K and ERa signaling (Figure 1B). ERα can rapidly activate PI3K through nongenomic mechanisms, either via RTKs or through direct interaction with the p85 regulatory subunit (26, 27). Conversely, PI3K/AKT signaling enhances ER transcriptional output via phosphorylation of ERa and its coregulators (28, 29).

PI3K signaling also converges with the MAPK/ERK pathway downstream of shared RTKs (Figure 1B), with reciprocal feedback loops shaping proliferative responses and therapeutic adaptation (30). Coupling of PI3K to mTOR and AMPK coordinates estrogen-driven growth with nutrient availability and cellular energy status, whereas interactions with the WNT/β-catenin, NF-κB, and TGF-β pathways further modulate differentiation, survival, and immune-related programs. Collectively, these interconnected signaling networks enable ER^^+^^ breast cancer cells to maintain transcriptional and metabolic plasticity and underlie adaptive resistance to both antiestrogen and PI3K-targeted therapies.

### Oncogenic PIK3CA alterations.

*PIK3CA* encodes p110α, which consists of five distinct domains (Figure 2A): the p85 adaptor-binding domain (ABD), the Ras-binding domain (RBD), the C2 domain, the helical domain, and the kinase domain (31). Its regulatory subunit, p85α, is also composed of five domains, referred to as SH3, breakpoint-cluster region homology (BH), N-terminal SH2 (nSH2), inter-SH2 domain (iSH2), and C-terminal SH2 (cSH2) (31).

Gain-of-function (hypermorphic) mutations in *PIK3CA* are among the most common alterations across tumor types (32). In breast cancer, the most common mutations (“hotspots”) are H1047R in the kinase domain, followed by E545K and E542K in the helical domain (33–35). Recent biochemical and molecular studies have revealed that different mutations activate PI3K via distinct mechanisms. Moreover, accumulating evidence indicates that a smaller but biologically important subset of *PIK3CA* alterations may exhibit neomorphic properties, including altered regulatory dependencies and mutant-specific signaling outputs that extend beyond simple amplification of PI3K signaling (7).

In the prevailing model for PI3K activation (Figure 2B), p85 couples with phosphotyrosine (PY) residues in RTKs, which releases the inhibitory nSH2 contacts with the p110α catalytic subunit (19). Notably, this conformational change is mimicked by the helical domain mutations (19). As a result, the exposed nSH2 domain binds with higher affinity to tyrosine residues in pYXXM motifs on activated RTKs and/or their adaptor proteins (36). Conversely, mutations in the C-terminus of the kinase domain, especially H1047R, are associated with increased membrane binding through a conformational change reorientating its Wnt-inhibitory factor (WIF) binding motif and with enhanced kinase activity due to the flexibility of the activation loop (37). Importantly, this conformational change exposes a cryptic pocket near position H1047 (37, 38), which has been exploited as a potential binding site for allosteric inhibitors (39, 40).

Aberrant activation of the PI3K pathway can occur through additional mechanisms, including amplification of oncogenic RTKs (41), genetic inactivation of tumor suppressors regulating the pathway (42), or stimulation by upstream growth factor receptors such as insulin receptor tyrosine kinase (INSR) and the related insulin-like growth factor 1 receptor (IGF1R) (41).

Finally, although deletions in *PIK3CA* are rare, most reported deletions cluster in a narrow region of critical residues within the C2 domain. Such deletions represent about 10% of all *PIK3CA* mutations, and disrupt binding with the iSH2 domain of p85a, thereby derepressing p110α activity (43, 44). Tumor-associated mutations in *PIK3R1* and *PIK3R2*, the genes that encode p85a and p85b, respectively, have also been described (45). By destabilizing the interaction with their corresponding p110 isoforms or by destabilizing PTEN, these mutations aberrantly activate PI3K and generate tumor cell dependence on PI3K signaling.

## PI3Kα inhibitor evolution: selectivity, tolerability, and precision oncology

The high prevalence of *PIK3CA* mutations across solid tumors and the identification of recurrent “hotspot” activating alterations established PI3Kα as a compelling therapeutic target. However, translating this biological rationale into clinical success has proved challenging, with decades of research resulting in only few PI3K pathway–targeting compounds approved by the US FDA. This limited therapeutic armamentarium largely reflects the central role of PI3K signaling in normal cellular homeostasis and physiology, such that its inhibition frequently leads to substantial, dose-limiting toxicities (DLTs) (46). Based on their mechanism of action, PI3K inhibitors can be broadly classified into (a) pan-PI3K inhibitors, (b) orthosteric PI3Kα inhibitors that directly bind the ATP-binding pocket of the p110α catalytic subunit and block its kinase activity, and (c) a newer class of allosteric PI3Kα inhibitors (Table 1 and Figure 3).

### First generation: pan-PI3K inhibitors.

Early compound development focused on pan-PI3K inhibitors with the goal of achieving total pathway blockade. These trials collectively failed to translate into clinical practice but provided critical lessons that fundamentally reshaped the development of subsequent PI3K-targeted therapies. Among the candidate compounds in this class, buparlisib was tested in patients with HR^^+^^/HER2^^–^^ breast cancer in the BELLE clinical trials (47, 48). Preclinical investigations had shown that compensatory PI3K crosstalk with ERα required a combination approach in HR^^+^^ breast cancers (49, 50). Thus, the selective ER degrader (SERD) fulvestrant was tested in combination with buparlisib, but the strategy showed an unfavorable risk-benefit ratio: a limited improvement in progression-free survival (PFS) was accompanied by serious adverse effects, such as altered liver enzymes, hyperglycemia, and neuropsychiatric symptoms (48, 51). Collectively, these clinical and preclinical findings highlighted two priorities for future drug development: improving isoform selectivity to enhance the therapeutic index and combining PI3K inhibitors with endocrine therapies to overcome adaptive signaling.

### Second generation: orthosteric PI3Kα-selective inhibitors.

The second generation of PI3K inhibitors consisted of compounds selectively targeting the PI3Kα isoform. These PI3Kα-selective agents demonstrated improved pharmacologic tolerability and on-target pathway suppression, resulting in meaningful clinical activity in patients with molecularly selected tumors (52, 53). The clinical activity of taselisib was evaluated in the SANDPIPER trial, which met its primary endpoint of improved PFS in patients with *PIK3CA*-mutant HR^^+^^/HER2^^–^^ breast cancer. However, the magnitude of benefit was modest, and treatment was associated with a high incidence of adverse events — including diarrhea, hyperglycemia, and colitis — leading to frequent dose reductions and treatment discontinuations (54). This toxicity profile may partly reflect the incomplete isoform selectivity of taselisib. Despite showing greater activity against mutant PI3Kα than against the wild-type enzyme, this drug also inhibits PI3Kβ, heavily expressed in the intestinal and colonic epithelium, and PI3Kγ and PI3Kδ, which are predominantly expressed in immune cells (54).

Alpelisib was the first PI3Kα inhibitor approved by FDA, following the results of the phase III SOLAR-1 trial (55). SOLAR-1 enrolled patients with HR^^+^^/HER2^^–^^ advanced breast cancer who had progressed on or after prior endocrine therapy. Patients were stratified in two cohorts based on *PIK3CA* mutation status and randomized to receive fulvestrant in combination with alpelisib or placebo. SOLAR-1 established *PIK3CA* mutation status as a predictive biomarker of response. While no difference in PFS was observed between treatment arms in the wild-type cohort, in patients with *PIK3CA*-mutated tumors, median PFS was 11 months in the alpelisib–fulvestrant group compared with 5.7 months in the control group (55). Median overall survival (OS) was 39.3 months in the alpelisib arm versus 31.4 months in the placebo arm, although this difference did not reach statistical significance (52). However, wide clinical use of alpelisib has been limited by metabolic toxicity, particularly hyperglycemia, and by the modest durability of response (34). Notably, SOLAR-1 completed patient enrollment before CDK4/6 inhibitors became the standard of care, therefore only 5.9% of patients in this trial had received a prior CDK4/6 inhibitor. To address this point, the phase II single-arm BYLIEVE and the phase III EPIK-B5 studies were designed, evaluating the efficacy of alpelisib in the post-CDK4/6 inhibitor setting (56, 57). In the BYLIEVE trial, in the patients receiving alpelisib median PFS was 8.0 months and median OS was 27.3 months (56). In the EPIK-B5, the median PFS was 7.4 months in the fulvestrant plus alpelisib arm versus 2.8 months in the fulvestrant plus placebo arm, while OS is not yet available (57). However, the emergence of AKT inhibitors, which have demonstrated clinically meaningful efficacy with a favorable therapeutic index in patients with *PIK3CA*/*AKT1*/*PTEN*-altered tumors after CDK4/6 inhibitors, may limit the clinical applicability of these findings (58, 59).

Inavolisib (GDC-0077) is a PI3Kα-selective, PI3Kb-sparing agent with improved pharmacologic properties and enhanced target engagement compared with alpelisib (53). Indeed, inavolisib also promotes degradation of the mutant p110α protein, potentially enabling prolonged suppression of oncogenic signaling (53). This dual mechanism enhances antitumor activity while minimizing off-target inhibition of other PI3K isoforms. Importantly, the rationale for sustained PI3K pathway suppression is further supported by the extensive functional crosstalk among ER, CDK4/6, and PI3K signaling networks (60, 61), which cooperatively drive endocrine therapy resistance. These observations provided the biological basis for combined targeting of all three pathways in the phase III INAVO120 trial (62), where the addition of inavolisib to palbociclib and fulvestrant significantly improved PFS compared with palbociclib and fulvestrant in patients with *PIK3CA*-mutated, endocrine-resistant HR^^+^^/HER2^^–^^ advanced breast cancer. Notably, the median PFS was 15 months in the triplet-combination arm versus 7.3 months in the control arm of fulvestrant plus palbociclib (62). There was also a statistically significant OS benefit of 7 months, the first to be shown in a phase III trial evaluating a PI3K inhibitor–based combination (62). Collectively, these findings suggest that simultaneous blockade of ERa, CDK4/6, and PI3K more efficiently eliminates pathway crosstalk, thus abrogating treatment resistance and disease progression. However, hyperglycemia, which can result from blockade of AKT2-mediated glucose uptake in muscle and adipose cells together with increased hepatic glucose production, was reported by approximately 63% of patients (all grades), of which 6.8% were grade ≥3 (62). Stomatitis/mucosal inflammation emerged as a notable toxicity with inavolisib, occurring at substantially higher rates than observed with alpelisib in the SOLAR-1 or BYLIEVE studies. This was primarily managed with dexamethasone mouthwash, with dose modifications required in a minority of patients. Despite the addition of a third agent, treatment discontinuation due to adverse events with the inavolisib triplet remained low and compared favorably with historical rates reported for alpelisib-based doublets (62).

### Allosteric mutant-selective PI3Kα inhibitors.

Therapeutic selectivity against mutant PIK3CA is intended to preserve antitumor efficacy while reducing on-target metabolic toxicities, particularly hyperglycemia. The improved tolerability, in addition to enabling deeper pathway blockade, potentially broadens the repertoire of combinations with other therapeutic agents, such as SERDs, CDK4/6 inhibitors, anti-HER2 therapies, and others.

Structure analyses have linked H1047R-*PIK3CA* to an altered conformation of the C-terminal tail of p110α (Figure 2B) (19, 20). Subsequent studies leveraged these structure differences to develop allosteric mutant-selective inhibitors (39, 40). Unlike earlier compounds that inhibit both wild-type and mutant PI3Kα with comparable potency, these next-generation molecules are designed to preferentially target oncogenic *PIK3CA*-mutant isoforms while sparing the wild-type enzyme. Several compounds are included in this category, such as zovegalisib (RLY-2608) (39), tersolisib (STX-478) (40), LOXO-783 (63), and OKI-219 (64). The clinical development of LOXO-783 was halted following the emergence of dose-limiting gastrointestinal toxicity, predominantly diarrhea, raising questions about its degree of selectivity against mutant PI3Ka (63). These findings also reinforce the need for strategies that optimize not only molecular selectivity, but also tissue exposure and dosing schedules.

Currently, zovegalisib is being investigated in combination with fulvestrant with and without CDK4/6 inhibitors in the phase I/II ReDiscover trial. This PIK3CA mutant selective inhibitor recently obtained Breakthrough Therapy Designation by the FDA. Encouraging data were presented at the American Society of Clinical Oncology 2025 meeting, indicating that, in patients with metastatic disease, the median PFS was 11.0 months when they were treated with fulvestrant plus zovegalisib in the second-line setting and 10.3 months in those treated beyond the second-line setting (65). The ongoing ReDiscover-2 Study aims to assess the efficacy and safety of zovegalisib plus fulvestrant versus the pan-AKT inhibitor capivasertib plus fulvestrant in patients with *PIK3CA*-mutant HR^^+^^/HER2^^−^^ breast cancer following progression on or after treatment with CDK4/6 inhibitors (NCT06982521). In parallel, tersolisib is being tested in the phase I/II PIKALO-1 trial (NCT05768139), including a first cohort where the treatment is administered as monotherapy in patients with advanced solid tumors; a second cohort of patients with HR^^+^^/HER2^^–^^ breast cancers is treated with tersolisib plus fulvestrant; and a third cohort is treated with endocrine therapy (aromatase inhibitors, fulvestrant, or imlunestrant) and a CDK4/6 inhibitor. The phase III PIKALO-2 trials will investigate endocrine therapy (aromatase inhibitors or fulvestrant) plus CDK4/6 inhibitors with tersolisib or placebo, both in treatment-naive, endocrine therapy–sensitive as well as pretreated endocrine therapy–resistant patients. The results of these trials are expected to better define the role of next-generation PI3Kα inhibitors within the evolving treatment landscape of HR^^+^^/HER2^^−^^ metastatic breast cancer.

## Mechanisms of resistance and biomarkers of response

The efficacy of targeted therapies is frequently limited by complex adaptive processes occurring both within cancer cells and in their interactions with the tumor microenvironment. Genetic alterations, signaling rewiring, and phenotypic plasticity collectively contribute to resistance and shape treatment sensitivity. A deeper understanding of the mechanisms by which cancer cells evade PI3K inhibition may inform the development of rational combination strategies as well as predictive biomarkers of response.

### Downstream PI3K pathway reactivation.

Resistance to PI3K inhibitors frequently converges on restoration of PI3K pathway signaling. Among these molecular alterations, loss of the tumor suppressor PTEN is one of the most frequent events, resulting in derepression and thus hyperactivation of PI3K/AKT signaling (Figure 4A) (66, 67). PI3K signaling in PTEN-null cancers has been shown to depend the p110β isoform (68). Recently, it was reported that PTEN loss promotes PI3Kβ phosphorylation and the subsequent formation of a signaling complex with ephrin receptor A2 (EPHA2) and SRC (69). This complex sustains PI3Kβ phosphorylation and drives oncogenic signaling through p-ERK/c-MYC and p-AKT (69). In this context, the FDA-approved SRC/EPHA2 inhibitor dasatinib was able to suppress tumor progression in mouse models. However, PI3Kβ inhibitors have demonstrated only modest efficacy across PTEN-null tumor models (70, 71), suggesting that pathway dependency cannot be fully explained by catalytic inhibition alone and prompting investigation into alternative mechanisms sustaining PI3Kβ signaling.

Another mechanism of resistance involves residual mTOR activity sustained by pyruvate dehydrogenase kinase 1 (PDK1) and serum/glucocorticoid-regulated kinase 1 (SGK1). SGK1, a downstream effector activated by PDK1, sustains residual mTORC1 activity by directly phosphorylating and inhibiting TSC2 (Figure 1A). This residual mTORC1 activity is sufficient to weaken the antitumor activity of PI3Kα inhibition. Thus, simultaneous suppression of both AKT and PDK1 is required to achieve complete mTORC1 inhibition and antitumor activity in tumors resistant to PI3Kα antagonists (72).

### RTK feedback and ERα reactivation.

Upon PI3K inhibition, reduced AKT kinase activity leads to decreased phosphorylation of FOXO3A, allowing its translocation to the nucleus (Figure 4B). In turn, nuclear FOXO3A can modulate transcriptional programs promoting adaptive survival and pathway rewiring, such as HER3 and other RTKs (73, 74), as well as expression of ERα and ERα target genes (75). In HER2-overexpressing tumors, PI3K inhibition triggers compensatory upregulation of HER3, which promotes the formation of HER2-HER3 heterodimers and enhances HER3 phosphorylation (73). Phosphorylated HER3 provides multiple docking sites for p85 resulting in partial restoration of PI3K pathway activity (76). Other RTKs, including IGF1R and INSR, are upregulated following PI3K inhibition, reflecting relief of negative feedback loops that normally restrain upstream signaling. Another mechanism of adaptive resistance involves ERα signaling (Figure 1B). PI3K inhibition is associated with increased ERa transcription (50) mediated by enhanced activity of KMT2D, a histone H3 lysine 4 methyltransferase (77). Toska and colleagues showed that, under basal conditions, AKT1 binds and phosphorylates KMT2D, attenuating its methyltransferase activity and ERα function, whereas PI3Kα inhibition enhances KMT2D activity resulting in an open chromatin state at ERa target loci (77).

### Metabolic adaptation.

Under physiological conditions, insulin and IGF-1 receptor signaling rely predominantly on the p110α catalytic subunit of PI3K to generate PIP3 and activate AKT-mediated glucose uptake and survival pathways. Consequently, pharmacologic inhibition of PI3Kα not only suppresses tumor-intrinsic signaling but also disrupts glucose homeostasis by impairing glucose uptake in insulin-responsive tissues, such as liver, muscle, and adipose tissue, and promoting hepatic gluconeogenesis, ultimately causing hyperglycemia (Figure 4C). This metabolic perturbation stimulates compensatory insulin secretion from pancreatic β cells that, in turn, reactivates INSR and IGF1R, which are ubiquitous in tumor cells, partially restoring PI3K pathway activity through p110α and/or alternative PI3K isoforms. Thus, this adaptive feedback loop attenuates pathway suppression and dampens the antitumor action of PI3Kα inhibitors (78). Moreover, hyperglycemia in patients is a DLT that results in dose reduction and treatment interruption (79), potentially contributing to treatment inefficacy. However, numerous preclinical studies have demonstrated that adaptive resistance can emerge despite near-complete pharmacologic inhibition of PI3K signaling under saturating experimental conditions (70, 78, 80). Therefore, adaptive rewiring represents an intrinsic response to pathway inhibition that is distinct from, although potentially exacerbated by, DLTs. Unless PI3Kα-mutant selective inhibitors could completely spare wild-type PI3Ka, this adaptive compensatory signaling may also occur in tumors treated with such drugs, impairing their full antitumor effect.

### Other compensatory signaling pathways.

Resistance to PI3K inhibitors may also happen through activation of compensatory pathways. Indeed, inhibition of PI3K can lead to increased RAS/RAF/MEK/ERK activity in HER2-overexpressing breast cancer cells (81), and vice versa, blocking MAPK can increase phosphorylated AKT levels (82). Crosstalk between the PI3K/AKT/mTOR (PAM) and MAPK pathways happens at multiple points of these networks (Figure 1). RAF is inhibited by AKT or the small GTPase Rheb (83, 84). TSC2, a repressor of mTORC1, is inhibited by ERK (85). In breast cancer cells, these interactions are further complicated by additional MAPK and PI3K crosstalk with ERα (see above). Preclinical studies have addressed the implications of this crosstalk by combining PI3K and MEK inhibitors, demonstrating synergistic effect but also major side effects (82, 86, 87). On a similar note, KRAS activation mediates resistance to PI3K inhibition (88). MAPK signaling mutations occur in approximately 10% of breast cancers, with the most frequent alterations being inactivating mutations in *NF1*, resulting in loss of the GTP-activating protein neurofibromin and subsequently activation of RAS, and activating mutations in *KRAS* (89), occurring particularly in endocrine-resistant tumors (90). As the predominant clinical use of PI3K inhibitors is in endocrine therapy–resistant tumors, these data warrant further clinical research with recently approved RAS inhibitors.

### Acquired genetic resistance.

Longitudinal plasma circulating tumor DNA (ctDNA) analysis from the phase I/II trial of alpelisib plus aromatase inhibitors showed that mutations in the DNA binding domain of *ESR1* (e.g., Y537S, D538G), the gene encoding ERα, expanded under treatment with antiestrogen therapy and PI3K inhibitors and that the presence of these mutations at baseline was significantly associated with lack of clinical benefit (67). The use of SERDs, active against *ESR1* mutations, may overcome this limitation, as suggested by the ctDNA analysis in the ReDiscover trial using zovegalisib (65). In addition to *PIK3CA* mutations and PTEN loss, PI3K signaling may be activated through *AKT1* mutations, present in approximately 3% of breast cancers. The most common mutation is *AKT1* E17K, which results in the protein being constitutively localized at the plasma membrane where it is activated by PDK1 and TORC2, while strongly binding PIP3 (91). As a result, AKT1 E17K is resistant to PI3K inhibitors (92, 93) but highly sensitivity to AKT inhibitors (94).

Although an infrequent event, PI3K activity can also increase via amplification or gain of *PIK3CA* copy number (95). In HER2^^+^^ breast cancer cells, high-level amplification of mutant *PIK3CA* was shown to cause resistance to the pan-class I PI3K inhibitor pictilisib (GDC-0941) (96). Amplified c-Myc has also been associated with resistance to PI3K inhibitors (97–99). A recent study in colorectal cancer cells showed that, upon treatment with PI3K inhibitors, an internal ribosome entry site within the MYC upstream region induces a FOXO3a-dependent transcriptional program that ultimately upregulates MYC, thus causing PI3K inhibitor resistance via enhanced autophagic flux (100).

Using a combination of structural modeling and free energy perturbation simulation, Varkaris and colleagues uncovered secondary mutations in the *PIK3CA* gene that promoted acquired resistance to PI3Kα orthosteric inhibitors (93). Notably, mutations in the W780 and Q859 positions, located within the PI3Kα ATP-binding site, confer resistance to orthosteric PI3K inhibitors, but this resistance can be overcome by allosteric PI3K inhibitors. The authors also found that some secondary *PIK3CA* mutations in *cis* with the primary mutation, e.g., E726K in *cis* with H1047R may decrease the effect of the allosteric inhibitor zovegalisib (93), while retaining sensitivity to orthosteric inhibitors (alpelisib, inavolisib).

Not all complex *PIK3CA* genotypes confer resistance. A study from Vasan et al. showed that in 12%–13% of cases, double *PIK3CA* mutants amplify signaling and enhance oncogenicity but also pathway dependence; such tumors retain exquisite sensitivity to alpelisib and inavolisib compared with single mutants. The double mutants generally include a major mutation in either the helical or kinase domain and a minor mutation in *cis* (e.g. E453, E726, M1043) (101). By contrast, mutations in *trans* (e.g. E726K/H1047R) are less sensitive than the mutations in *cis*, responding similarly to the single mutants (101). This study highlights limitations of the established practice of sequencing only certain variants or exons, which underestimates the frequency of double mutants at <1%, without position information (102).

## Clinical perspectives and future directions

As the field continues to evolve, the future clinical role of PI3K inhibitors will be determined not only by improvements in their molecular target selectivity, but also by development of drugs against other key components in this signaling pathway and rational combinations that abrogate adaptive mechanisms of resistance.

As the therapeutic landscape in HR^^+^^ breast cancer becomes more complex, treatment selection and sequencing strategy of PI3K inhibitors will be increasingly challenging. PI3K inhibitor efficacy is limited in tumors with PTEN loss-of-function and activating AKT1 mutations, suggesting that patients whose tumors harbor these alterations require alternative therapeutic strategies. One such strategy is use of pan-AKT inhibitors such as capivasertib, which has demonstrated clinical benefit in tumors harboring activating AKT1 mutations and PTEN loss, supporting its use as a biologically rational option for these molecular subsets as well as tumors relying on wild-type PIK3CA, potentially ahead of PIK3CA mutant–selective inhibitors with a more limited coverage of the PI3K/AKT pathway.

Indeed, AKT small-molecule antagonists have also been developed to target tumors that depend on aberrant PI3K signaling. The ATP-competitive pan-AKT inhibitor capivasertib has received FDA approval, in combination with fulvestrant, for patients with ER^^+^^/HER2^^–^^ breast cancer harboring mutations in *PIK3CA*, *AKT*, or *PTEN* based on results of the CAPItello-291 trial (58, 103). This study showed a longer median PFS in patients treated with fulvestrant plus capivasertib versus fulvestrant plus placebo (7.2 months versus 3.6 months; HR, 0.60) (58). Although capivasertib has similar side effects as alpelisib, e.g., rash and diarrhea, it is associated with a lower incidence of hyperglycemia, which may be attributable to its intermittent dosing schedule (4 days on/3 days off). Further, by blocking a key signaling hub downstream of all PI3K isozymes, AKT inhibitors may suppress a broader range of PI3K-driven signaling than PI3Ka inhibitors. Currently, capivasertib is increasingly used in the second-line setting. The future role of capivasertib in the treatment landscape of tumors with somatic alterations in the PI3K pathway may in part depend on the results of the ongoing ReDiscover-2 trial comparing fulvestrant plus capivasertib versus fulvestrant plus zovegalisib (NCT06982521).

Recently, the therapeutic landscape has further expanded with the development of the PAM pathway inhibitors, agents capable of targeting multiple alterations in the pathway — including nodes that mediate resistance to more selective PI3K inhibitors. The small-molecule PAM inhibitor gedatolisib was shown to be more effective than “single-node” PAM inhibitors, such as alpelisib, capivasertib, and the mTORC1 inhibitor everolimus, in tumor cells with either mutated or wild-type PAM signaling (104). Gedatolisib was associated with manageable side effects, possibly because of the reported lower IC__50__ of the compound in cancer cells compared with nontumor cells and pulsatile pathway inhibition as a result of weekly i.v. administration, potentially decoupling efficacy from metabolic toxicity through this dosing strategy rather than molecular selectivity (104). Gedatolisib was tested in combination with endocrine therapy with or without palbociclib in a phase I/Ib trial in patients with or without *PI3KCA* mutations, where it was associated with a reduced rate of hyperglycemia compared with other single-node PAM inhibitors (105). The phase III trial VIKTORIA-1 evaluated the efficacy of the triplet gedatolisib/palbociclib/fulvestrant and the doublet gedatolisib/fulvestrant versus fulvestrant alone, in patients with wild-type *PIK3CA* (study 1) or versus fulvestrant/alpelisib in patients with *PIK3CA*-mutant cancers (study 2). Enrolled patients had HR^^+^^/HER2^^–^^ advanced breast cancer, which had progressed during or after aromatase inhibitors plus CDK4/6 inhibitors (105). While the results for the *PIK3CA*-mutant cohort are forthcoming, the results from study 1 were reported (106), and the median PFS for the triple combination was 9.3 months versus 2.0 months in the fulvestrant monotherapy group, while fulvestrant plus gedatolisib median PFS was 7.4 months. The reduced risk of death in patients treated with the triplet combination versus single agent fulvestrant is so far the largest difference reported in this patient population (106).

If the results in the *PIK3CA*-mutant cohort in the VIKTORIA study are positive, multiple implications arise for future drug development. First, PAM inhibitors seem to work in absence of *PIK3CA* mutations. This finding is consistent with the BOLERO-2 trial results, where everolimus plus exemestane was active independently of presence of any mutation in the PI3K pathway (107). On the other hand, gedatolisib was shown to be less effective against PTEN-null cancers in preclinical studies (108), but these cases were not excluded from the VIKTORIA trials. In any case, the identification of other compounds that show clinical activity irrespective of PI3K/AKT pathway mutation status would imply the need for better therapeutically predictive biomarkers of pathway dependence and thus sensitivity to pathway-targeted inhibitors (109, 110). Indeed, integration of genomic, transcriptomic, epigenomic, and proteomic data generated through large-scale initiatives, such as The Cancer Genome Atlas and Clinical Proteomic Tumor Analysis Consortium, has shown that mutational status alone does not always correspond to aberrant PI3K pathway activation, highlighting the complementary value of phosphoproteomic and transcriptomic profiling for identifying tumors with functional PI3K dependence (109–113).

Another emerging therapeutic strategy involves disrupting oncogenic protein-protein interactions, which are increasingly recognized as druggable therapeutic targets (114, 115). One such example is the interaction between PI3Kα and RAS family members through the RBD. Compounds designed to disrupt this interaction have been referred to as “PI3K-KRAS breakers.” Mutations such as T208D and K227A in the PI3Kα RBD disrupt the interaction between PI3K and KRAS (116). Based on this observation, a new compound that phenocopies the effect of the PI3Kα T208D-K227A mutant, BBO-10203, has been developed (117). BBO-10203 is specific to PI3Kα and exhibits no affinity toward PI3Kβ, PI3Kδ, and PI3Kγ. BBO-10203 is effective in decreasing AKT phosphorylation in *PIK3CA/KRAS*-mutant cells, while PTEN-deficient models appear largely unresponsive to the treatment. HER2-amplified tumors showed a greater-than-expected sensitivity to BBO-10203, although the underlying mechanism remains unclear. In HER2-amplified tumors, PI3Kα activation is not strictly dependent on canonical RAS activity (118), suggesting that this sensitivity may be mediated through effects on noncanonical RAS-related proteins, including RRAS, RRAS2, and MRAS (117). Further investigation of this mechanism is warranted; nevertheless, the observed activity in HER2-amplified models suggests a potential therapeutic role for this compound in this subset of patients. Notably, these antitumor effects were achieved without the development of hyperglycemia, highlighting a unique therapeutic property, as insulin signaling mediates glucose uptake through mechanisms that are independent of RAS-driven PI3Kα activation.

Identification of effective pathway inhibitors has important implications for optimizing treatment sequencing. *PIK3CA* mutations associated with resistance to the orthosteric inhibitor alpelisib do not appear to impair sensitivity to the allosteric inhibitor zovegalisib (93). AKT1-E17K mutations have been found in patients resistant to either allosteric or orthosteric PI3K inhibitors (92, 93), but those patients are sensitive to AKT inhibitors (94). In this context, PAM pathway inhibitors are likely to be of particular interest. More broadly, a deeper understanding of resistance mechanisms may inform the development of rational combination strategies and improve patient selection for clinical trials. A clear example of this approach is the exclusion of patients with *PTEN*-null tumors from trials evaluating PI3Ka inhibitors. Whether patients harboring tumors with activating RAS mutations should also be excluded is not clear. Achieving these advances will require well-designed ancillary studies to generate the necessary biological and clinical insights, ultimately enabling more durable and profound responses while carefully balancing treatment-related toxicities.

Another promising area for PI3K inhibition is the neoadjuvant setting. As *PIK3CA* mutations are truncal events present from diagnosis, targeting this pathway early in the disease course is biologically compelling. A previous study adding alpelisib to endocrine therapy did not improve objective response rates, pathological complete response rates, or Ki-67 suppression compared with endocrine therapy alone, suggesting that the impact of PI3K inhibition may differ between early and metastatic disease (119). However, the results of simultaneous blockade of ERa, CDK4/6, and PI3K in the INAVO120 trial suggest that early use of this combination in some patients with high-risk disease is worthy of clinical testing. In this context, the role of inavolisib in combination with letrozole and palbociclib in endocrine therapy–sensitive, *PIK3CA*-mutant breast cancer is being investigated in the INAVO123 trial (NCT06790693). In the future, replacing inavolisib with a *PIK3CA*-mutant selective inhibitor may improve the tolerability and efficacy of this combination.

*PIK3CA* mutations are highly prevalent in lobular breast cancers, often at higher frequency than in ductal carcinoma (120, 121). While no PI3K inhibitor trial has been specifically designed for this subtype, there is increasing interest in targeting the PI3K pathway in studies enriched for this population. In this context, an ongoing trial (NCT06607757) is evaluating the AKT inhibitor capivasertib in combination with fulvestrant versus fulvestrant alone in primary high-risk lobular breast cancer.

Finally, in addition to patient selection defined by molecular criteria, it is important to consider the differences in trial eligibility criteria, especially in terms of HbA1c thresholds and acceptable glycemic control. Earlier trials of alpelisib, such as SOLAR-1 (52), largely excluded patients with diabetes or prediabetes, whereas more recent studies of PI3K pathway inhibitors have adopted more permissive eligibility criteria, allowing patients with well-controlled diabetes. In particular, PIKALO-1 (investigating tersolisib/STX-478) is the most permissive trial to date, with 54% of patients enrolled with baseline prediabetes or diabetes, providing a more clinically representative assessment of hyperglycemia risk (122). Despite this broader eligibility, metabolic toxicity remained limited: among patients with prediabetes or diabetes, rates were 37% for any-grade hyperglycemia and only 1% for grade ≥3 events. No grade 3 hyperglycemia occurred among patients with normal baseline glycemic parameters. Conversely, patients with type 1 diabetes or uncontrolled type 2 diabetes are excluded from the VIKTORIA-1 and ReDiscover2 trials and, in the latter, even patients with type 2 diabetes requiring medication are excluded.

In conclusion, PI3K/AKT pathway inhibition is a key therapeutic strategy in breast cancer, with drug development still evolving across clinical disease contexts and molecular subgroups. Ongoing clinical and translational efforts will be crucial to improve patient selection, overcome resistance mechanisms, and maximize clinical benefits. In particular, enhancing biomarker-driven approaches and integrating novel combinations will be central to unlocking its full therapeutic potential.

## Conflict of interest

NU declares personal fees from Pfizer, Lilly, and Gilead Sciences. CLA reports grants from Pfizer and Eli Lilly and scientific expert advisory activities for Lilly and Pfizer.

## Funding support

This work is the result of NIH funding, in whole or in part, and is subject to the NIH Public Access Policy. Through acceptance of this federal funding, the NIH has been given a right to make the work publicly available in PubMed Central.

- National Cancer Institute grants P30 CA142543 and R01 CA273246.
- Breast Cancer Research Foundation.

## Figures and Tables

**Figure 1:**
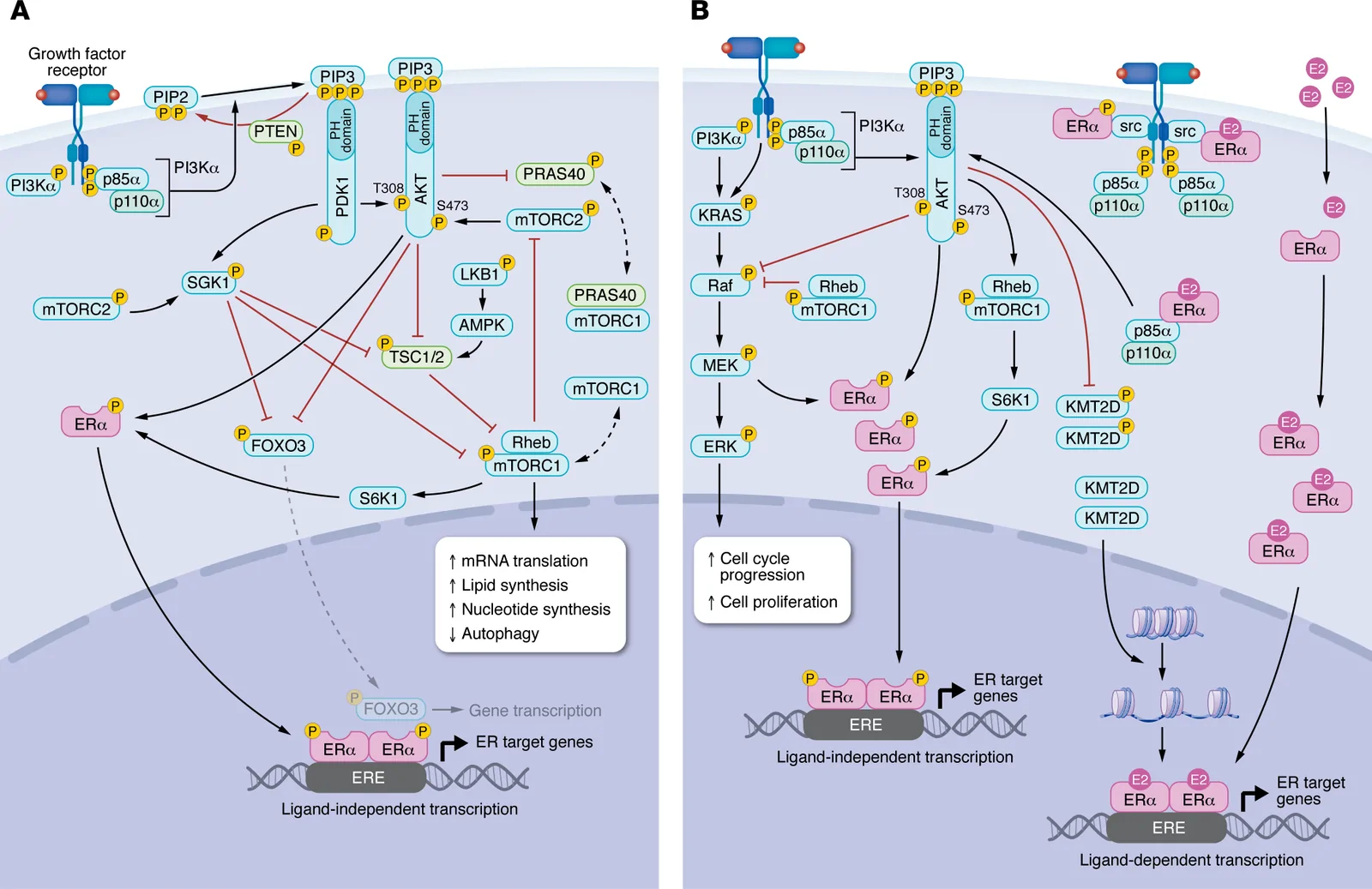
PI3K signaling and crosstalk with ERα and MAPK pathways. (**A**) RTK activation recruits class IA PI3K (p85α/p110α), promoting conversion of PIP2 to PIP3 at the plasma membrane. PIP3 recruits PDK1 and AKT, enabling AKT phosphorylation by PDK1 (on Thr308) and by mTORC2 (on Ser473). AKT inhibits the TSC1/2 complex, relieving suppression of Rheb and activating mTORC1, which drives mRNA translation, lipid and nucleotide synthesis, and inhibits autophagy through S6K1 signaling. PDK1 can activate SGK1, which can also phosphorylate and inhibit TSC2, thereby sustaining AKT-independent residual mTORC1 activity. Both AKT and SGK1 phosphorylate FOXO3, promoting its cytoplasmic sequestration and functional inactivation. When not phosphorylated, FOXO3 translocates to the nucleus where it induces transcriptional programs that compensate for PI3K inhibition. Additional regulatory inputs from LKB1/AMPK signaling and PTEN-mediated PIP3 dephosphorylation are shown as negative modulators of the pathway. (**B**) Crosstalk between PI3K signaling, MAPK, and ERα happens at multiple nodes. PI3K can activate MAPK via interaction with KRAS. Activated AKT can phosphorylate ERα, activating ligand-independent transcription of ER-target genes. Ligand activated and/or phosphorylated ERα can bind RTKs via adaptor proteins (e.g., Src) and favor phosphorylation of RTKs at tyrosines in their C-terminus. These residues provide docking sites for p85 and activation of p110α, the catalytic subunit of PI3K.

**Figure 2:**
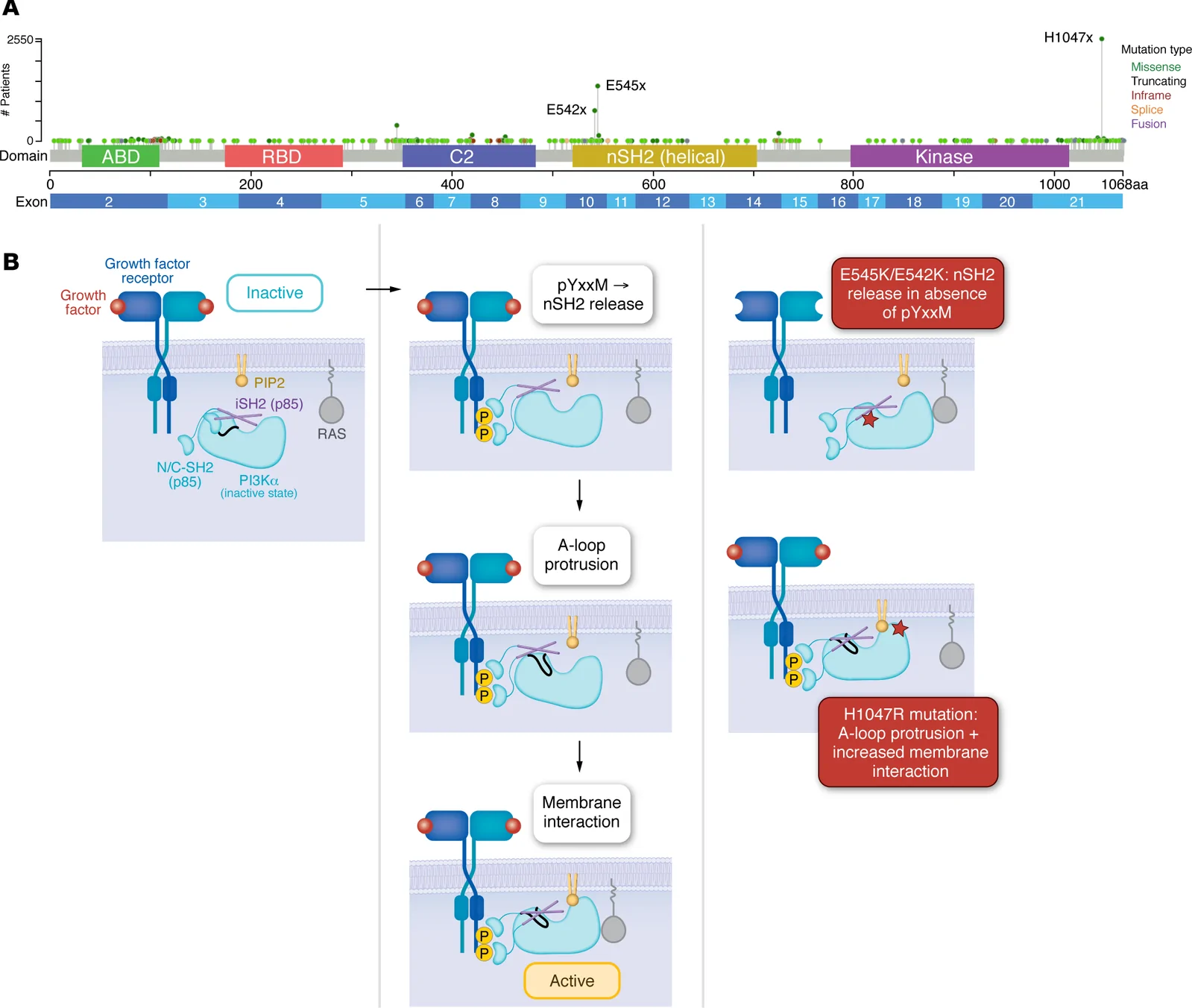
Landscape of PIK3CA mutations and their predicted functional consequences. (**A**) Lollipop plot of the *PIK3CA* gene, illustrating exons, domain organization, and position of point mutations. The GENIE cohort v19.0-public was interrogated for PIK3CA mutation frequency, type, and position in the breast cancer cohort (*n* = 6,812 specimens/6,044 patients) (5). PIK3CA (p110α) comprises five functional domains: the adaptor-binding domain (ABD, in green), which mediates interaction with the regulatory p85 subunit; the Ras-binding domain (RBD, in red), which binds active RAS proteins and contributes to enzyme activation; the C2 domain (in blue), involved in membrane association and interaction with p85; the helical domain (in yellow), which regulates kinase activity through interactions with the p85 nSH2 domain; and the kinase domain (in purple), which contains the catalytic site responsible for phosphorylation of PIP2. (**B**) Schematic representation of PI3K activation and oncogenic PI3Kα mutations. In the basal state (left), PI3K is maintained in an inactive conformation through inhibitory intramolecular interactions within the regulatory p85 subunit (N- and C-terminal SH2 domains) and the catalytic p110α subunit. Upon growth factor stimulation, activated RTKs recruit p85 via SH2-phosphotyrosine interactions, thus releasing the nSH2-mediated inhibition of p110α. This allows A-loop exposure and engagement of PIP2 in the membrane, resulting in enzymatic activation and conversion of PIP2 to PIP3. RAS-GTP can further enhance membrane recruitment and activation. Oncogenic mutations promote constitutive PI3K activation through distinct mechanisms. Helical domain mutations (E542K/E545K) disrupt the inhibitory interaction between the p85 nSH2 domain and p110α, enabling PI3K activation even in absence of RTK activation. In contrast, the H1047R mutation enhances membrane interaction and A-loop protrusion, increasing catalytic activity.

**Figure 3:**
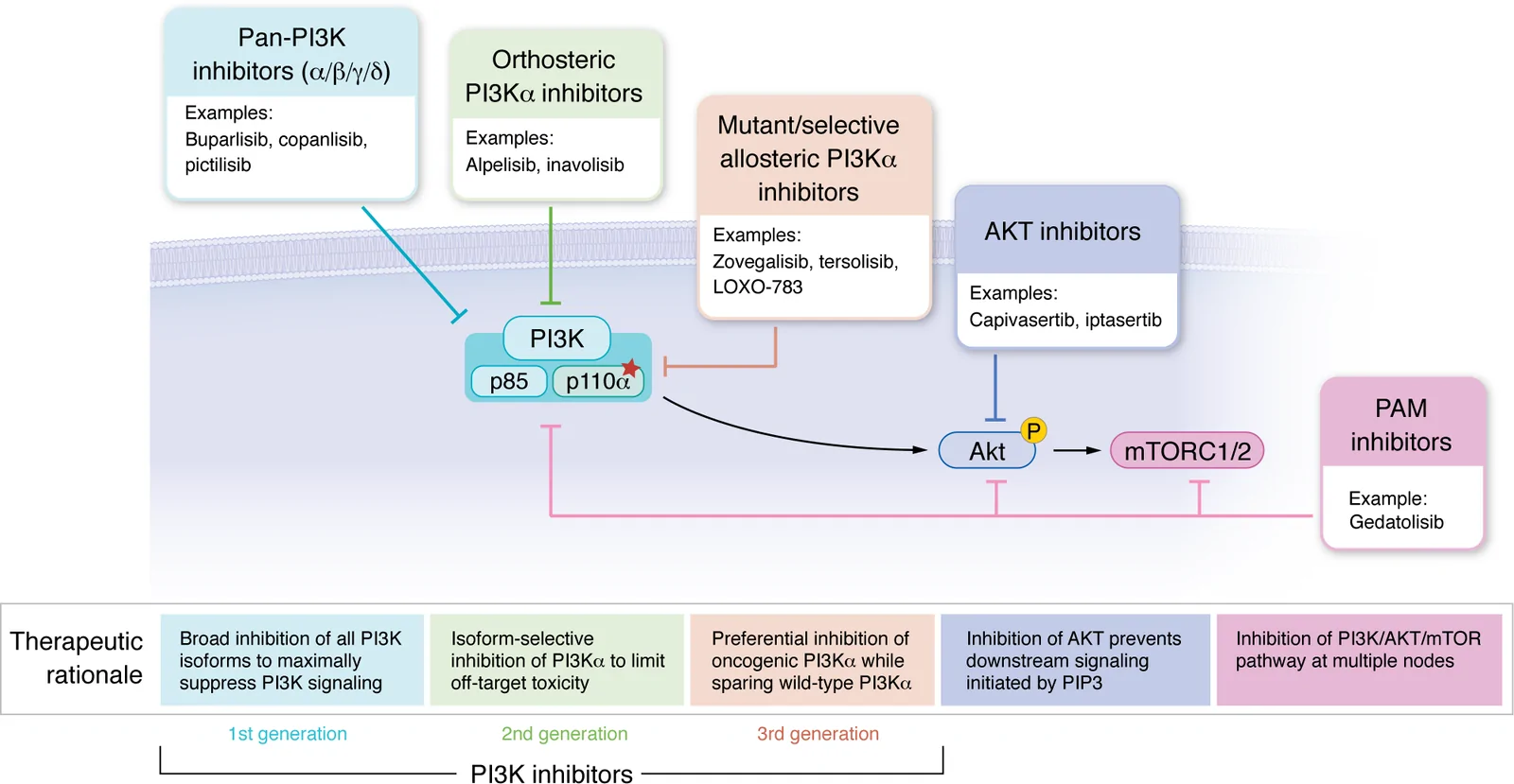
PI3K/AKT/mTOR signaling pathway inhibitors and their sites of action. Therapeutics have been developed targeting PI3K, AKT, mTOR, or all three (PAM inhibitors).

**Figure 4:**
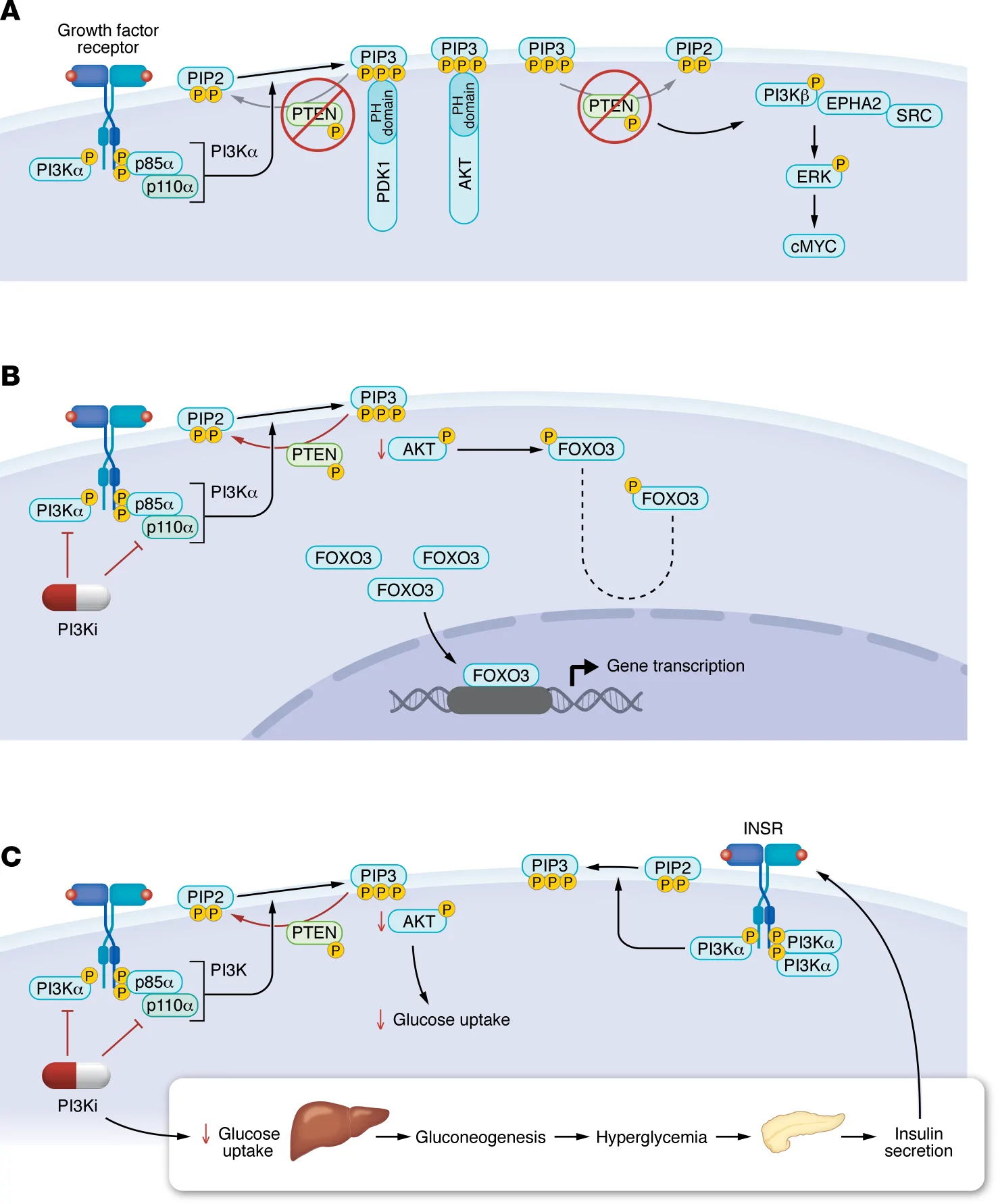
Representative mechanisms of adaptation and resistance to PI3K inhibitors. (**A**) Downstream pathway reactivation. Loss of PTEN causes increased levels of PIP3 and thus hyperactivation of PI3K signaling. As a result, PI3Kβ is phosphorylated/activated by forming a complex with EPHA2 and SRC that, in turn, induces cMYC via ERK. (**B**) Upstream RTK feedback. Treatment with PI3K inhibitors reduces AKT activity and inhibits FOXO3 phosphorylation in the cytoplasm. Unphosphorylated FOXO3 translocates to the nucleus where it induces transcription of RTK genes, thus favoring RKT-mediated feedback reactivation of PI3K and dampening inhibition of PIP3 formation. (**C**) Metabolic adaptation. Inhibition of PI3K impairs glucose uptake in host tissues (muscle, adipose tissue, liver) via modulation of AKT2 activity. Reduced glucose uptake by the liver activates gluconeogenesis, further favoring hyperglycemia. This stimulates insulin secretion by the pancreas resulting in activation of INS/IGFR1 receptors in cancer cells, partial reactivation of PI3K signaling, and dampening inhibition of PIP3 formation.

**Table 1:**
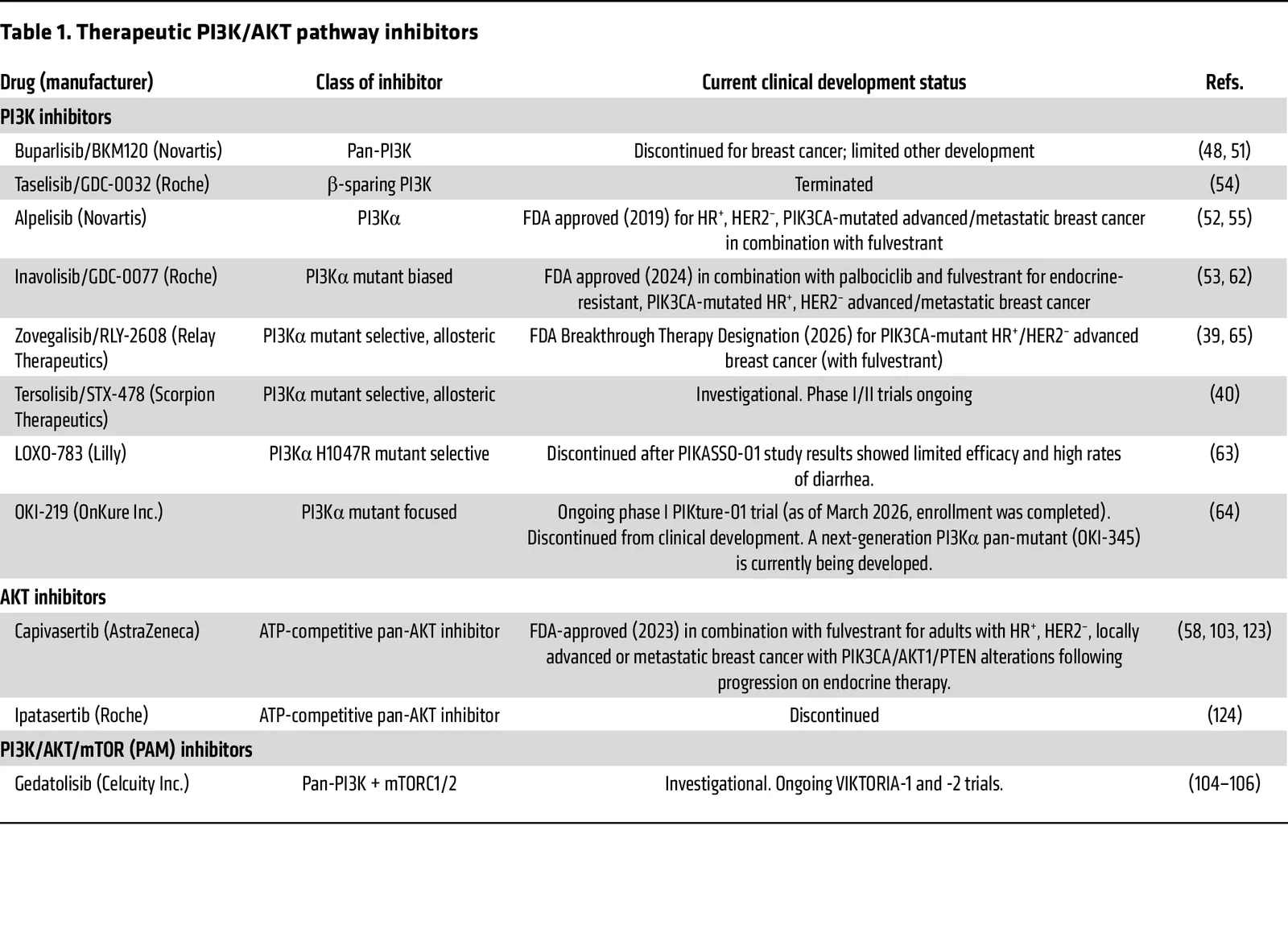
Therapeutic PI3K/AKT pathway inhibitors

## Version 1 10/15/2026

Electronic publication
